# Progress in Surgery

**Published:** 1895-05-04

**Authors:** 


### PROGRESS IN SURGERY.

### SURGERY OF THE INTESTINES.

(*Continued from page* 65.)

**Cancer of the Colon.**—Where there is the least doubt as to position of the tumour, Bland Sutton[11] advises a preliminary exploration in the linea alba before resorting to colotomy. The chief advantages of this method are: (1) It will occasionally prevent the surgeon opening the colon unnecessarily. (2) The obstruction may be due to an ovarian or uterine tumour, easily removable. (3) The obstruction may be due to adhesions or dissemination of cancer. (4) It will prevent the surgeon in some cases opening the left and then the right loin. Demons[12] expresses an opinion that a surgeon is justified in attempting, oftener than is actually done, the radical cure of cancer of the colon. At present he is disposed to give the preference to the operation of resection in two stages. It presents the great disadvantage, however, of condemning the patient to a very troublesome infirmity for some time, not to speak of the danger associated with a second operation. It is, therefore, to be hoped that the radical cure in one sitting may be so perfected as to justify us in abandoning all other procedures. He reports a successful case of radical cure by the operation *à deux temps*, as also does H. Littlewood.[13] Magnes and Lilienthal[14] report a case in which the operation of resection of the carcinoma of the colon was done at one sitting with the aid of Murphy's button.

**Resection of the Cæcum.**—Sendler[15] reports a case in which this operation was done in a young woman aged twenty-two years for obstruction due to carcinoma. He says, resections of the cæcum are rare, Sachs having been able to find but thirty cases. The chief indications for the operation are carcinoma and tuberculosis, and in a few instances on account of invagination. Magill[16] has given the subject much consideration, and he finds that resection of the ileo-cæcal coil has been performed 102 times. Twenty-nine of these cases terminated fatally, placing the general mortality at 28·43 per cent. He says that excision of the cæcum is justifiable in primary neoplastic tumours, in tuberculosis, or inflammation at the ileo-cæcal coil, in persisting fistula following appendicitis, in invagination in the region of the cæcum, and in irreducible cæca. The greater part of the mortality after excision of the ileo-cæcal coil is directly imputable to the insufficiency of the sutured intestine. The length of time required for operation is maximum for sutures. With the exception of a fault of too little incision, not a reproach is found for resection followed by ileo-colostomy with absorbible plates or with the anastomotic button, these last methods having so much time-saving advantage. Lichtenstein has shown from a study of 154 cases that the ileo-cæcal coil is occupied by primary tumour as frequently as the entire small intestine, and more than any other intestinal region beyond the immediate vicinity of the rectum.

**Intestinal Fistula and Artificial Anus** are best treated, according to Senn,[17] by a preliminary transverse suturing of the intestinal opening (including all the tunics of the bowel) as a prophylactic measure against infection. After the lumen of the intestine, with its contents, is shut off from the field of operation, the surface is again disinfected. The next step in the operation consists in including in two elliptical incisions the margins of the abdominal opening and the scar tissue in its vicinity. The peritoneal cavity is opened by a straight incision extending downwards from the lower angle of the two incisions. The bowel is detached from the abdominal wall and drawn forwards into the external incision. The strip of skin and scar tissue is carefully trimmed away from the bowel with scissors, and the provisional sutures are then buried by a row of Lembert's sutures. The bowel is then replaced in the abdominal cavity and the external wound closed. Chaput[18] records thirty-five cases of artificial anus and stercoral fistula which he has treated. He says artificial anus can be treated by four methods: (1) By the application of enterotomy, followed by obliteration of the fistula. He prefers Richelot's method of getting rid of the spur—that is section between two pairs of forceps and immediate suture—where the spur is long and thin. (2) Resection, which as a general rule is contra-indicated. (3) Longitudinal enterorraphy, when enterotomy is for some reason contra-indicated. (4) Entero-anastomosis, followed by ligature of the two ends between the point of anastomosis and the stercoral aperture. It is indicated when the intestine is very friable at the seat of the artificial anus, or when there is a considerable constriction of the bowel in the neighbourhood of the external aperture, and also when the inferior end is obliterated at the level of the artificial anus.

**Strangulated Diaphragmatic Hernia** is the subject of an article by Rochard,[19] who asks, when the diagnosis is made for which laparotomy is necessary, ought reduction to be made by the abdominal or the transpleural route? Reduction is not always easy through the abdomen, and using a bistoury to free the bowel in the midst of coils of intestine is dangerous. The writer then advises the transpleural route, which, he says, is easy, but he has only performed it on the dead body. The patient is placed in the left lateral decubitus, with a cushion under the right flank, in order to make the left costal flank project. A U-, T-, or H-shaped incision four and a-half to six inches in length is made at the level of the ninth rib. A musculo-cutaneous flap is then raised, the ninth rib is divided, and may be excised or raised with the deep soft parts. The pleura is then opened, the lung retracts by itself, and the diaphragmatic arch is seen, upon which it is easy to operate. The herniotomy is conducted as usual, the sac being opened, &c. The radical cure can then be done as elsewhere; the sac is excised, the edges of the opening are freshened, and a row of sutures comprising the muscular tissue and the pleura is inserted. If there is not sufficient space, the tenth rib can be divided. The pleura is cleaned very carefully, and the wound in the thoracic wall closed. The abdominal wound, which has during these procedures been closed with a sponge, is now sutured.

[11] Clinic. Journ., Aug. 15th, 1894, p. 258. [12] Med. Week, Oct. 26th, 1894. [13] Lancet, Oct. 13th, 1894, p. 853. [14] New York Med. Journ., Sept. 1st, 1894. [15] Amer. Journ. of the Med. Sci., Oct., 1894, and Münch. Med. Woch., 1894, No. 1. [16] Annals of Surgery, Dec., 1894, p. 642. [17] Amer. Journ. of Obstet., Sept., 1894, p. 323. [18] Brit. Med. Journ., Dec. 15th, 1894, and Arch. Gén. de Méd., Sept. and Oct., 1894. [19] Quarterly Med. Journ., Oct., 1894, p. 70.

